# Therapeutic Efficacy of Oncolytic Viruses in Fighting Cancer: Recent Advances and Perspective

**DOI:** 10.1155/2022/3142306

**Published:** 2022-07-22

**Authors:** Ghazal Asadi Garmaroudi, Farzaneh Karimi, Leila Ghanbari Naeini, Pajman Kokabian, Nozar Givtaj

**Affiliations:** ^1^School of Medicine, Tehran University of Medical Sciences, Tehran, Iran; ^2^Behbahan Faculty of Medical Sciences, Behbahan, Iran; ^3^School of Medicine, Gulf Medical University, Ajman, UAE; ^4^School of Medicine, Shahid Beheshti University of Medical Sciences, Tehran, Iran; ^5^Rajaie Cardiovascular Medical and Research Center, Iran University of Medical Science, Tehran, Iran

## Abstract

Immunotherapy is at the cutting edge of modern cancer treatment. Innovative medicines have been developed with varying degrees of success that target all aspects of tumor biology: tumors, niches, and the immune system. Oncolytic viruses (OVs) are a novel and potentially immunotherapeutic approach for cancer treatment. OVs reproduce exclusively in cancer cells, causing the tumor mass to lyse. OVs can also activate the immune system in addition to their primary activity. Tumors create an immunosuppressive environment by suppressing the immune system's ability to respond to tumor cells. By injecting OVs into the tumor, the immune system is stimulated, allowing it to generate a robust and long-lasting response against the tumor. The essential biological properties of oncolytic viruses, as well as the underlying mechanisms that enable their usage as prospective anticancer medicines, are outlined in this review. We also discuss the increased efficacy of virotherapy when combined with other cancer medications.

## 1. Introduction

Cancer leftovers one of the world's utmost common causes of mortality. The necessity for novel treatment options is becoming increasingly critical as the global cancer incidence continues to increase. Despite the availability of numerous therapeutic methods for cancer treatment, such as surgery, radiation, and chemodrugs, the risk of recurrence remains significant. Immunotherapeutic approaches for cancer treatment have become increasingly popular in preclinical research and clinical practice over the last decade [1]. Traditional oncological strategies are aimed at removing or killing tumor cells directly.

On the other hand, immunotherapy is performed to improve the immune system's ability to destroy cancer cells, leading to tumor regression, establishment of antitumor immune memory, and, finally, long-lasting reactions. This can be accomplished through various strategies such as monoclonal antibodies, cancer vaccines, and immune checkpoint inhibitors [2]. It was discovered in the early 1900s that natural viral infection caused tumor regression, which sparked an interest in using viruses to treat cancer. However, because of concerns about viral pathogenicity and toxicity, this technique was ruled out. Oncolytic viruses (OVs) are a new generation of viruses created as a result of recent developments in genetic engineering technologies that assure their safety and potency [3].

Information about the immune response to new or even old viruses is constantly changing and needs to be updated [4], because viruses' characteristics change due to geographical and natural interactions [5]. Scientists discovered that some viruses could naturally kill tumor cells in the last century. OVs were generated from wild-type or naturally attenuated virus strains during this time, leaving a lot of space for development in terms of safety and antitumor effects. OV-mediated cancer virotherapy has emerged as a new and successful cancer treatment strategy. Many OVs have been used, and numerous viruses have been studied for cancer therapy [6]. OVs are cancer-targeting viruses that can be native or recombinant. The viruses kill cancer cells in their last stages of replication by lysis or by stimulating an antitumor immune reaction, consequently diminishing impairment in healthy organs [7]. The antitumor efficacy of OVs is based on a number of processes that include the natural interactions between viruses, cancer cells, and the immune system. Another benefit of OVs for cancer therapy is that they may be utilized to impress gene expression in the tumor microenvironment, which either enhances the OVs' ability or boosts the immune system's antitumor arm [8].

The fundamental knowledge of viruses' anticancer properties is rapidly converted into feasible therapy alternatives for aggressive cancers, which is a fascinating phase in the evolution of the OV field. The main goal of this review was to evaluate the monotherapy of OVs in comparison with other therapeutic approaches and highlight the importance of combination therapy because of insufficient antitumor response during OV monotherapy. Additionally, we describe how OVs are important in cancer diagnosis and treatment. Also, the synergistic efficacy of OVs in combination with targeted therapy, radiation, chemotherapy, and immunotherapeutic drugs is described in this review.

## 2. Nature of Oncolytic Viruses

More than a century has passed since the idea of utilizing viruses to cure cancer was first proposed. A middle-aged lady with a hematologic cancer was described to have had her tumor shrink owing to influenza as early as 1904. Later, in 1912, Italian researchers demonstrated that rabies vaccination might help eliminate cervical cancer, launching the OV treatment idea and a series of similar studies [9]. Researchers undertook several clinical experiments utilizing wild-type viruses to treat cancers in the 1950s and 1970s. Still, due to the virus's inability to adequately regulate its pathogenicity, the OV fell to second position in treatment of cancer. Genetic engineering technology did not allow for the modification of a virus's genome until the 1980s, which resulted in the introduction of genetically engineered weakened and highly selective viruses. Knocking down thymidine kinase in the gene-altered human herpes simplex virus I (HSV-1) could prevent glioma in mice, extend animal longevity, and be exceedingly safe, according to preclinical animal studies published in 1991. Onyx-015, a genetically engineered adenovirus, was approved for phase I clinical studies in 1996 [10, 11]. Although China granted a license for the altered adenovirus H101 in 2005, its clinical efficacy has yet to be confirmed outside China [12]. Talimogene laherparepvec (T-VEC) was authorized for commercialization by the Food and Drug Administration (FDA) in October 2015. T-VEC was authorized for marketing in Europe and Canada in 2016, indicating that OV technology for cancer treatment has progressed.

**Figure 1:**
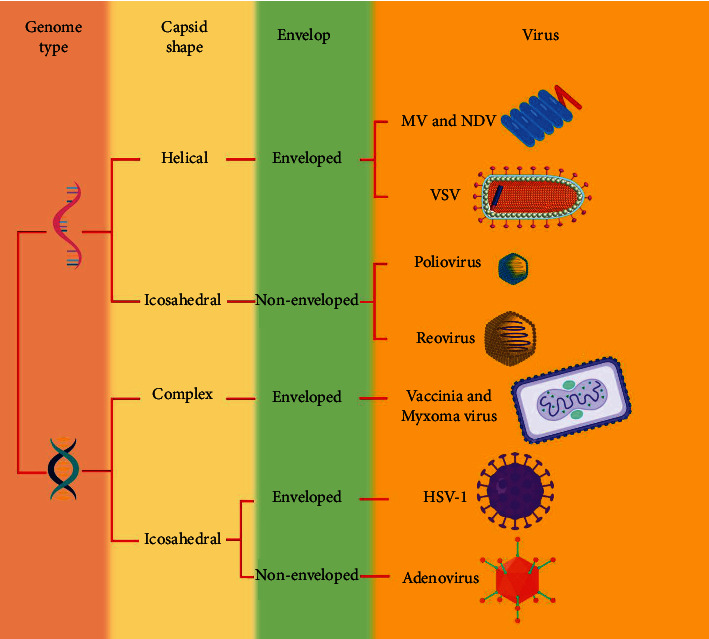
OVs as cancer therapy. In order to limit their pathogenicity, viruses that enter human cells need to be genetically engineered to be suitable for oncolytic virotherapy.

Three OV products have been authorized for commercialization, while six more are presently being experienced in phase III clinical studies [13]. Over standard tumor immunotherapies, OVs have a variety of advantages, including high killing efficacy, precision targeting, little adverse reactions or drug resistance, and relatively inexpensive [3, 14]. Another important advantage of OVs is their success rate. With the growth of novel models and approaches for virus replication in the 1950s and 1960s, there were numerous challenges to developing viruses with greater tumor specificity. Still, several researchers stopped working on development due to poor results. Some of the first human clinical trials with oncolytic viruses to treat advanced cervical cancer began in 1956. However, research in this subject was restricted for several years due to technological restrictions. Oncolytic virotherapy was nearly abandoned after the 1960s due to a lack of clear and encouraging outcomes from clinical studies; molecular methods were not as advanced at the time; therefore, oncolytic virotherapy was at risk of being abandoned totally. Now that genetic engineering has progressed so far and so quickly, interest in oncolytic virotherapy has been revived. A new generation of oncolytic virotherapy has emerged as a result of improved virology knowledge and experience with viruses in cancer gene therapy [15, 16]. So, in comparison to surgical therapy, chemoradiotherapy, and targeted therapy, oncolytic virotherapy is a potential cancer therapy (Figure 1).

Due to the particular cytokines that malignant cells produce, they are more susceptible to OV infection. Tumor-driver mutations, on the one hand, have been proven to augment viral selectivity in tumor cells [17, 18]. Besides, numerous tumor cells sustained preferential virus multiplication, probably due to a deficiency of antiviral type I interferon signaling [19, 20]. Aside from this, OVs vary in size and complexity, ranging from parvovirus H1 (5 kb linear, single-stranded DNA) to vaccinia (190 kb) and HSV1 (152 kb), which might lead to variances in the virus's capacity to infect cancer cells [21, 22]. To induce carcinogenesis, tumor cells impair antiviral responses; this permits viruses to be engineered to cause cancer cells to proliferate and lyse while triggering apoptosis in healthy cells. Although OVs can enter normal cells, they cannot actively reproduce in them since the cells would die, which prevents the virus from spreading to additional surrounding normal cells. On the other hand, cancer cells that suppressed p53 will retain vulnerability to viral effects, allowing the virus to propagate to neighboring cancer cells [23].

The use of OVs is a revolutionary addition to the anticancer therapeutic arsenal. The idea that OVs could be used as therapeutics arose from reports of spontaneous tumor regression in response to naturally occurring viral infections [24, 25]. Despite the abundance of naturally occurring OVs, there has recently been a surge of attention in genetically modifying viruses in order to provide potential cancer therapies [14, 26]. OVs operate by specifically targeting and killing tumor cells while also activating and developing anti-tumor immunity [27, 28]. This dual strategy of activity permits for direct local antitumor reactions (leading in tumor regression), as well as provoking the immune system [24, 29].

## 3. How Immune System Is Involved in Oncolytic Virus Therapy

**Figure 2:**
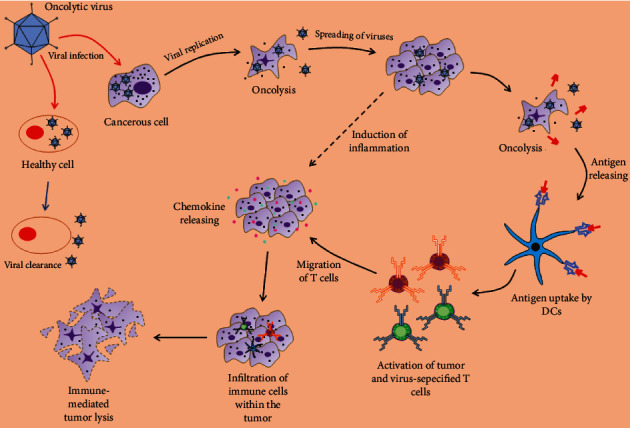
The interaction between OVs and the immune system. OVs can only multiply in cancer cells and not in normal cells, causing the tumor mass to lyse. However, OVs can also upregulate the immune system.

Tumors are described as an immunosuppressive milieu in which the immune system is suppressed to prevent any response to cancerous cells. The transfer of OVs into the tumor causes immunity and produces a powerful and durable response (Figure 2). This process is facilitated by both innate and adaptive immune responses [30–32].

Antigen-presenting cells (APCs), especially dendritic cells (DCs), have the great potentiality to present exogenous antigens on major histocompatibility complex (MHC) class I. Oncolytic reovirus infection in a mouse model, for example, boosted MHC class I, TAP-1, and TAP-2 expression, which was not experienced in the control cells [33]. In a separate investigation, adenovirus caused DCs to secrete IFN-*γ*, which prompted cancer cells to provoke PA28. This protein activates proteasomal cleavage to generate MHC class I antigens. This process resulted in elevated cytotoxic T lymphocyte lysis of infected cancer cells [34–36].

Additionally, oncolytic Newcastle disease virus (NDV) caused consistent overexpression of MHC class I in both control and infected cells, according to Zamarin et al. This was likely triggered by augmented type I IFNs [37]. A similar process was noticed by adenovirus armed with an IFN-*β* transgene in a lung cancer model [38].

Natural killer (NK) cells are innate immune system components that serve as central regulators and have a powerful tumor cytolytic activity [39]. Some lines of research have revealed that OVs and NK cells have a cross-talk in cancer immunotherapy. NK cells are vital immune regulators in oncolytic virotherapy. In an experimental procedure of oncolytic NDV combined with immune checkpoint inhibitors, Zamarin et al. noticed that NK cell depletion could remarkably restrict their therapeutic impact, implying that these cells are important [37]. On the other hand, OVs could boost NK cell proliferation and activity. By stimulating DC in vitro, oncolytic reovirus can improve NK cell function [40]. Additional experiment in immune-competent melanoma mouse models uncovered that oncolytic vesicular stomatitis virus stimulated IL-28 release, which supported NK cell activation [41].

Tumor-associated macrophages (TAMs) are hopeful oncology targets due to their responsibilities in the tumor microenvironment as tumor supporters and blockers of antitumor immunity [42]. As indicated by oncolytic paramyxovirus infection of macrophages, OVs act as major immunological stimuli and are advantageous in switching the phenotype functions of macrophages [43]. TAMs may develop an antitumor phenotype in response to oncolytic paramyxoviruses, according to findings from a recent study, to increase the antitumor impact of oncolytic virotherapy via tumoricidal pathways [44]. Furthermore, Saha et al. observed that triple combination therapy boosted macrophage infiltration and M1-like polarization, facilitating glioblastoma eradication [45]. Furthermore, intratumoral G47 administrations significantly reduced M2 macrophages. In contrast, elevated M1 macrophages and NK cells, according to Sugawara et al. These data suggest that G47 could be effective in the treatment of gastric cancer [46].

As a type of early myeloid cells, myeloid-derived suppressor cells (MDSC) are increased in a variety of diseases, including cancer, and have the ability to suppress immune reactions [47]. MDSCs have been shown to limit the success of cancer immunotherapy; thus, therapies targeted at these cells could be favorable [48]. In an experiment, it was demonstrated that oncolytic reovirus constrains the immune-suppressive activities of MDSCs through the Toll-like receptor 3 signaling pathway, and this action leads to reovirus-mediated tumor regression. They found that the suppressive function of MDSCs on T cell proliferation was noticeably decreased after administration of reovirus into tumor-bearing mice [49]. Also, Eisenstein et al. suggested that viral transduced MDSCs can shift from the protumor functional M2 phenotype to antitumor [50].

OVs have long been known to be one of the utmost powerful mediators of cytokine responses in the tumor microenvironment and tumor-bearing hosts [51]. A state of significant immunosuppression is common in the tumor microenvironment. Cancerous cells overexpress cytokines to limit normal antitumor immune reactions. Tumor-released cytokines and chemokines also contain those indorsing development and vascularization [27]. Infected cells and resident and infiltrating immune cells release cytokines, as well as chemokines (RANTES, MIP-1*α*/*β*), shifting the balance of pro- and anti-inflammatory mediators inside the tumor microenvironment [52]. These agents facilitate the recruitment of cytokine-secreting immune cells with extra effector roles, in addition, to direct antiviral and immunoregulatory effects. Infiltrating immune cells' effector functions are improved by a viral infection and the accompanying localized inflammation, which counteracts tumor-induced immunosuppression and facilitates the formation of antitumor immunity [53].

Lymphocytes detect certain antigens and initiate a predetermined response against them. Immune activation during OV therapy has the ultimate goal of activating T cells against tumor antigens. T cells have the ability to detect tumor peptides and give long-term protection. This implies that the primary tumor and metastatic sites will be affected. Besides, the patients will be protected even if they relapse due to T cells' capability to develop memory. As a result, to prevent lymphocyte activation, tumor cells frequently try to escape from the immune system. OV treatment can cause a cytokine storm, which leads to lymphocyte recruitment and the breakdown of the immune-suppressive environment [54]. Ribas et al. proposed that intratumoral injection of an oncolytic virus designed to recruit immune cells could boost anti-PD-1 immunotherapy by altering the tumor microenvironment in the injected lesions and promoting CD8+ T cell infiltration [55].

Furthermore, a new survey showed that oncolytic virotherapy could boost the antimyeloma T cell reaction, resulting in long-term tumor control in some cases. They reported that after virotherapy, multiple myeloma patients treated with oncolytic measles virus experienced a considerable rise in the percentage of circulating CD3+ and CD8+ T cells [56]. Various mechanisms, including induction of type I IFN signaling and chemokine secretion in response to viral antigens, can support the fact that OVs increase T cell trafficking and infiltration into tumor milieu [57]. In addition, immune cells attack the tumor site after the antigen is detected, and cancer cells that are not infected with OVs are also destroyed [58]. Furthermore, cytotoxic T cells' local production of perforins and granzymes successfully eliminates surrounding malignant cells, including those lacking antigen expression or altered antigens [59, 60].

OVs have the ability to elicit immunogenic cell death (ICD) in the same way as some standard anticancer therapies, including chemotherapy and radiation therapy, do [61, 62]. For instance, in orthotopic mouse glioma models, NDV immunotherapy has been shown to increase calreticulin translocation to the cell surface and extracellular accumulation of high mobility group box 1 and a tumor-specific immune response and long-term tumor regulation [63, 64]. In addition, measles and coxsackievirus B3 can cause the secretion of similar dangerous signal molecules, which cause infected cells to activate ICD in vitro, attracting a large number of immune cells to the tumor microenvironment. In summary, OVs stimulate the immunogenic death of tumor cells, resulting in the secretion of soluble antigens and inflammatory factors that support the stimulation of effector immune cells [65, 66].

## 4. Oncolytic Viruses in Cancer Diagnosis and Treatment

At this time, all types of advanced imaging technologies for tumor diagnosis, particularly Computed tomography and Magnetic resonance imaging, play an indispensable role in the precise positioning and local invasion evaluation of tumors. However, early diagnosis of primary tumors and small metastases remains a challenge, necessitating the development of imaging equipment with greater sensitivity and precision. Effective tumor imaging using OV has gotten a lot of attention in recent years. OVs carrying specific genes can infect and proliferate in tumor cells while also expressing the luciferase reporter gene and the human Na+/I-symporter gene [67], and we can notice gene expression products, to acquire molecular imaging [68]. One of OV's applications in precise tumor imaging was fluorescence imaging [69]. The green fluorescent protein, which is derived from marine invertebrate organisms, can be used to monitor tumor behaviors [70].

Oncolytic viruses, based on growing body of preclinical and clinical evidence, could be a particularly successful new cancer therapy [71, 72]. Nowadays, herpes viruses, adenoviruses, coxsackieviruses, poxviruses, polioviruses, measles viruses, reoviruses, and Newcastle disease viruses are some of the OVs under study for cancer therapy [73]. OVs' anticancer properties are derived from a variety of cancer-killing pathways. The virus's direct oncolysis of tumor cells, which is frequently a combination of apoptosis, necrosis, pyroptosis, and autophagic cell death, is the first pathway. One of these stands out for a specific OV. The second mechanism is OV antiangiogenesis and antivasculature, which results in apoptotic and necrotic death of uninfected cells in animals and humans [74, 75]. The final part is to use triggered innate and tumor-specific immune cells to cause cytotoxicity in cancer and stromal cells. Antitumor immunity contributes to the removal of uninfected cancer cells in primary and metastatic nodules, as well as the control of dormant micrometastases [76–78]. Some OVs have been genetically modified to express proapoptotic or tumor suppressor genes, which boost immune reaction and promote anticancer actions [79] and P53, or other family members are introduced as the most common cases. P53 mutations can be seen in a wide range of cancers, and delivering these characteristic into the cancer cell can improve OV efficiency [80, 81]. Given the importance of angiogenesis in tumor activity, another anticancer approach is genetic amplification of viruses to encode antiangiogenic transgenes or direct destruction of tumor vascular endothelial cells [80]. To achieve this goal, a variety of viruses have been armed with the vascular endothelial cell growth inhibitor, anti-VEGF single-chain antibody, or VEGF promoter-targeting transcriptional repressor, aiming to prevent neovascularization and stimulate apoptosis in endothelial cells [82–85].

It is presented that the oncolytic adenovirus CD55-Smad4 was effectively developed and successfully repressed colorectal cancer cell proliferation in vivo and in vitro. CD55-Smad4 stimulated the caspase signaling pathway, resulting in colorectal cancer cell apoptosis. Also, by overexpressing Smad4 and suppressing the Wnt/-catenin/epithelial-mesenchymal transition (EMT) signaling pathway, the oncolytic adenovirus dramatically reduced colorectal cancer cell motility and invasion [70]. Ye et al. found that oncolytic NDV infection of lung cancer cells activates the secretion of ICD determinants such as ecto-CRT, HMGB1, and HSP70/90. They indicated that this OV therapy stimulates autophagy-mediated ICD in lung cancer cells [86]. In addition, a combination of two newly developed recombinant oncolytic adenoviruses expressing mK5 or MnSOD therapeutic genes might dramatically decrease gastric cancer development by apoptosis induction, implying that it could be used to treat gastric cancer [87]. In the case of hepatocellular carcinoma, through its direct oncolytic effects and induction of ICD, a recombinant vesicular stomatitis virus and NDV vector offered a very promising vector platform [88]. T-Vec, a herpes virus that was successfully evaluated in a phase III study in melanoma and was licensed for clinical use by the FDA in 2015, is at the forefront of this field [89].

## 5. Combination of Cancer Treatment Strategies with Oncolytic Virotherapy

OVs have multiple anticancer mechanisms that act directly or indirectly on tumors, making virotherapy an appropriate therapeutic option for cancer therapy. Moreover, OVs exhibited remarkable combined platforms because of their engineering practicality and proven safety profiles. In fact, throughout the last few decades, various combination approaches for natural or engineered OVs have been examined in the lab and clinical trials (Table 1) [60].

Immune checkpoint inhibitors (ICI) are a frequent supplementary therapeutic approach for OV. OV enables the immune cell infiltration into the tumor milieu, and ICIs stop suppressing infiltrated immune cell function [90, 91]. The usage of OVs expressing miniantibodies and single-chain variable fragments against checkpoints has newly been revealed to inhibit checkpoints locally in the tumor microenvironment with lower side effects [92, 93]. Many clinical trials are now evaluating the combined effect of ICI and OV, the consequences of which mainly recommend that in order to reach a better result, ICI should be administrated after the start of OV responses. OV raises the success of tumorinfiltrating leukocyte (TIL) and CAR-T cell therapy. By changing the tumor matrix and boosting chemokines, OV can improve TIL and CAR-T cell entry to the tumor [94]. The release of IL-15, TNF-*α*, and IL-2 from OVs in the tumor microenvironment supports the in situ proliferation and stimulation of TILs and improves tumor reaction to CAR-T cell therapy [94, 95]. The combination of OV with DC vaccines also increases the effectiveness of DC vaccines by shifting the tumor microenvironment immunosuppressive situations [96]. OVs could be applied as tumor vaccines in order to boost the immune responses against recognized tumors or even stop tumor recurrence. The key function of such OV-based tumor vaccines is endorsing the APC maturation to stimulate proper antitumor immune reactions [97].

**Figure 3:**
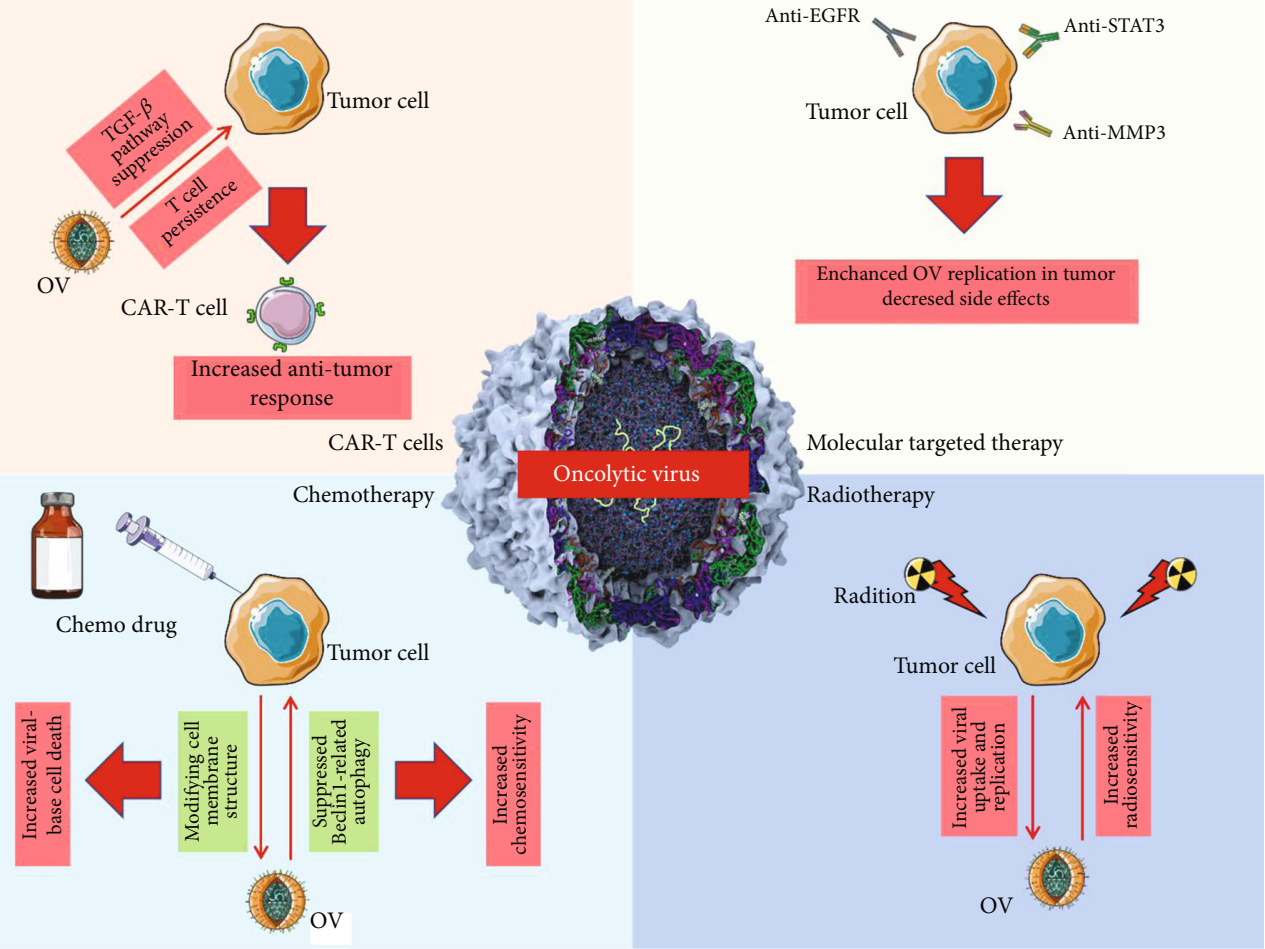
The combination of OV therapy with other therapeutic approaches. The combination of OV therapy with molecular targeted therapy, chemotherapy, radiotherapy, and CAR-T cell therapy could significantly enhance the treatment output and support the sensitivity of tumor cells to common therapeutic factors.

The synergistic efficacy of OVs in combination with targeted therapy, radiation, chemotherapy, and immunotherapeutic drugs is described in the following section (Figure 3).

### 5.1. Combining Oncolytic Virotherapy with Molecularly Targeted Therapy

Small molecular drugs and biological antibodies are being developed to alter abnormal signaling pathways and protein expression in cancer. Combining these targeted medications with OVs seems to be a hopeful therapeutic option [98]. For example, in human pancreatic cancer xenograft models, researchers tested the combination effect of HF10, a herpes simplex virus type 1 (HSV-1) mutant, with Erlotinib, an epidermal growth factor receptor tyrosine kinase inhibitor. According to the researchers, Erlotinib augments the oncolytic activity of HF10 by virus persistence within subcutaneously administered tumors [99]. In addition, NSC74859, a particular inhibitor of signal transducer and activator of transcription 3 (STAT3), has been shown to decrease hepatocellular carcinoma cell growth effectively and can be coupled with vesicular stomatitis virus-based oncolytic virotherapy. In the presence of this inhibitor, the vesicular stomatitis virus not only maintained its strong oncolytic effect in target cancer cells, but its harmful potential in healthy hepatocytes and neurons was significantly reduced [100]. Other findings revealed that matrix metalloproteinase-3 (MMP3) generated by tumor cells is essential for tumor cell proliferation and migration. In mouse colon cancer models, targeting MMP3 with diverse approaches in combination with OV therapy significantly improved tumor therapy, suggesting its potential as a new therapeutic opportunity for cancer therapy [101]. Furthermore, according to the findings of a recent study, the second mitochondrial activator of caspase-mimetic chemical and oncolytic virus therapies work together to drive CD8+ T cell responses against tumors via different actions [102].

### 5.2. Oncolytic Virotherapy in Combination with Radiotherapy

Combination therapies utilizing OVs and radiation have demonstrated encouraging results in vitro and in vivo, with each therapy operating synergistically with the other to eliminate tumor cells [103, 104]. Although the underlying mechanism of this synergistic antitumor effect is unclear, numerous explanations have been proposed: oncolytic viruses may support tumor cell radiosensitivity, making them more sensitive to radiation cytotoxicity, or radiation may induce cell death by elevating viral uptake and replication [105]. The in vitro and in vivo impact of Lister strain oncolytic vaccinia virus combination with radiation in head and neck cancer models was revealed in a study by Mansfield et al. They reported that combining radiation with chemotherapy resulted in increased cell death, activation of caspase activity, and enhanced long-term regression rates [103]. Gao et al. also found that colorectal cancer cells infected with the TRAIL-Armed oncolytic adenovirus and exposed to radiation therapy were destroyed in a dose-dependent manner. They observed that colorectal cancer cells treated with individual oncolytic adenovirus and radiation had a viability of 45 percent and 73 percent, respectively; however, when cells were given the combined treatment, viability decreased to 10 percent [106].

Moreover, it is confirmed that the use of radiotherapy increased adenovirus dl520 cytotoxicity with improved viral release, replication, and output. The oncolytic function of dl520 and radiation prevents glioblastoma development [107]. OBP-301 is introduced as a novel adenovirus that replicates in specific tumors and causes death of a range of tumor cells, the safety of this virus established in patients with solid tumors. Tanabe et al. performed a phase I clinical study to assess OBP-301's safety, tolerability, and efficacy when used in conjunction with radiation. They determined that the combination of OBP-301 with radiotherapy was well tolerated and capable of providing significant therapeutic advantages in esophageal cancer patients [108].

### 5.3. Oncolytic Virotherapy in Combination with Chemotherapy

The use of virotherapy in combination with chemotherapeutic drugs is a promising strategy. OVs have been studied as accompanied by various conventional chemotherapeutics, which have been classified according to their mechanism of action [109, 110]. Alkylating agents, DNA intercalators, nucleotide analogues, cellular cytoskeleton modifiers, and cytostatic agents are some of the most commonly used medications [111, 112]. Regardless of their mechanism of action, chemotherapy medications affect all rapidly dividing cells, not just tumor cells. As a result, chemotherapy is frequently linked to high toxicity levels and serious adverse effects [113]. OVs have a higher level of tumor-specificity than chemotherapeutic medications because to both their natural preference for tumor cells and specificity-enhancing genetic changes. Since the antitumor activities of OVs and chemotherapeutic medicines are mediated through separate mechanisms, many researchers suggest that when they are used together, they may operate synergistically [114].

According to the findings of a recent study, oncolytic adenovirus Ad11, in combination with Cisplatin, has a synergistic impact in treating osteosarcoma cells. The reason for this could be that after treatment with oncolytic adenovirus Ad11, the Beclin1-related autophagy pathway is suppressed, resulting in decreased Cisplatin resistance and increased cell death [115]. In addition, a phase I/II research looked into the use of the oncolytic virus T-VEC in combination with radiotherapy and cisplatin to treat patients with head and neck malignancies. These clinical trials have revealed that oncolytic virus and chemotherapy combination reduces cancer development and relapse and supports overall survival [116]. In the case of laryngocarcinoma, in vitro, the combination of oncolytic virus ZD55-TRAIL and Doxorubicin inhibited cell development more effectively while having minor negative effects on normal cells.

Furthermore, oncolytic virus-mediated tumor cell death was boosted by chemotherapy drugs [110]. Mao et al. observed that Docetaxel combination with oncolytic adenovirus armed with IL-24 (ZD55-IL-24) dramatically elevated caspase-3 and caspase-8 levels compared to a single treatment in vitro and in vivo. Besides, the TUNEL assay revealed that tumor tissues treated with Docetaxel and ZD55-IL-24 had a considerably higher apoptotic rate than tumor tissues treated with individual Docetaxel or ZD55-IL-24 [117]. In addition, combining oncolytic virus SG511-BECN with Doxorubicin promotes cytotoxicity in human chronic myeloid leukemia cells synergistically. Chemotherapy drugs may make cell membranes more vulnerable to infection by oncolytic adenovirus. The uncovering of signaling proteins implicated in the autophagic and apoptotic pathways demonstrated that SG511-BECN operates in combination with Doxorubicin to trigger autophagy-targeted death in chronic myeloid leukemia cells [118].

### 5.4. Oncolytic Virotherapy in Combination with CAR-T Cell Therapy

Chimeric antigen receptor T (CAR-T) cells and OVs have been successful cancer immunotherapy factors [119]. In addition, there have been a few successful cases of integrating virotherapy technology with CAR-T cell immunotherapy treating malignancies so far [120]. In a triple-negative breast cancer model, researchers tested the combined usage of CAR-T and oncolytic viral therapy. They revealed that an oncolytic adenovirus targeting TGF-*β* could destroy cancer cells and immediately provide noticeable antitumor reactions. In contrast, the antitumor function decreased at a late phase, though long-term antitumor benefits were shown with CAR-T cell therapy and a greater antitumor reaction may be identified at a late stage. Notably, oncolytic adenovirus targeting TGF-*β* and CAR-T cells in combination therapy had the maximum therapeutic benefits and antitumor immune responses [121]. Huang et al. also developed an oncolytic adenovirus containing IL7 and integrated it with B7H3-targeting CAR-T cells to investigate its efficiency in treating glioblastoma. They found that the combined use of them presents synergistic antitumor impact in vitro and in vivo by supporting T cell persistence, resulting in longer survival of tumor-bearing mice [122].

### 5.5. Combining Oncolytic Virotherapy with Checkpoint Inhibitors

The immune checkpoint inhibitors (ICIs) bind to immune checkpoint receptors and inhibit immunological inhibitory signals, known as immune checkpoint blockade (ICB). Combining OVs with ICIs is one of the most prominent ways to improve their effectiveness since this combination lessens the tumor immunosuppressive environment [123, 124]. In addition, the infection generated by OVs stimulates an anticancer immune reaction, supporting the efficiency of ICIs, which in the process disturbs the ligand-receptor interaction of tumor cells exposing T cells to attack [90, 125]. As a result, anticancer research has focused on combining OVs with ICB to improve consequences, and clinical trials have evaluated a variety of combination treatments [91].

Wang et al. developed manipulated oncolytic virus that coexpressed a PD-L1 inhibitor and GM-CSF. Their findings revealed that this engineered oncolytic virus could induce neoantigen-specific T cell responses on cancer cells and immune cells via the likely synergistic activity of viral replication, GM-CSF activation, and PD-L1 inhibition, implying that new oncolytic immunotherapy could be developed [126]. Also, a research group engineered an HSV-1-based oncolytic virus and examined its antitumor function in combination with pembrolizumab in humanized PD-1 knockin mice bearing nonimmunogenic B16-F10 melanoma. Their findings revealed elevated CD8+ and CD4+ T cell recruitment, IFN release, and PD-L1 expression in cancer, which resulted in mice's overall survival [127]. In a triple-negative breast cancer model, the therapeutic efficacy of oncolytic poxvirus CF33-hNIS-F14.5 combined with an anti-PD-L1 antibody was assessed. Immune modulation was stronger in mice treated with both the virus and an anti-PD-L1 antibody. While the individual drugs CF33-hNIS-F14.5 and anti-PD-L1 antibody had no substantial antitumor effect, the two agents together had a considerable antitumor impact, with 50% of mice exhibiting complete tumor regression when both treatments were administered intratumorally [128]. Besides, intratumoral administration of OVs results in immunological alterations in the local tumor milieu, including increased secretion of proinflammatory chemokines and cytokines, as well as immune cell activation, which supports the probability of refractory carcinomas responding to PD-1/PD-L1 inhibitors and lessens cancer development when combined treatment is used [129–131].

In addition to combining OVs with PD-1/PD-L1 inhibitors, combining OVs with CTLA-4 inhibition is a promising approach [60]. CTLA-4 is upregulated by OVs, attempting to make tumors susceptible to CTLA-4 inhibition. According to Zamarin et al., CTLA-4 was overexpressed after infection with NDV. However, a study of the immunological classification of tumor lesions reported that using NDV and CTLA-4 inhibitors together resulted in a significant increase in the ratio of effector T cells to Treg cells and a higher rate of stimulated immune cells [37]. It was established that combining anti-PD-L1 and anti-CTLA-4 with rAd.GM (oncolytic viruses containing the GM-CSF gene) boosted the anticancer effects in triple-negative breast cancer through regulating the tumor microenvironment [132]. A study considered the usefulness of G47 (an oncolytic herpes simplex virus type 1) combined with ICIs. The combination of G47 and anti-CTLA-4 antibodies was proven to effectively recruit effector T cells into the tumor while reducing regulatory T cells. Additionally, these types of combinations remarkably resulted in elevated expression of several genes associated with lymphoid lineage, inflammation, and T cell stimulation [133].

T cell immunoglobulin and ITIM domain (TIGIT) are currently studied as one of the most potential immune-checkpoint options. Compared to vaccinia virus alone (without anti-TIGIT insertion), intratumoral injection of vaccinia virus-anti-TIGIT boosted antitumor activity in some mice, including subcutaneous tumor models. The vaccinia virus modified with anti-TIGIT is an efficient method for oncolytic immunotherapy because it combines viral oncolysis with intratumoral production of immune checkpoint antibodies [134].

## 6. Conclusion and Prospects

Rapid advancements in molecular biotechnology have allowed researchers to design new ways to use the immune system to treat cancer. Furthermore, OV treatment is still evolving, and we now have a far better grasp of how they work. During last two decades, genetic engineering has facilitated the rapid progression of OVs, enabling even potentially harmful viruses to be adjusted for cancer treatment. They can affect the tumor's local immunological milieu in addition to lysing cells as part of viral replication. OVs have been noticed to disrupt the immune-suppressive milieu in tumors, allowing immunotherapeutics to work more effectively.

Appropriate drug delivery has been one of the most difficult obstacles to effective oncoviral therapy. The bioavailability of systemically injected oncolytic viruses is quite low. Furthermore, even when the virus is delivered intravenously, the attenuated virus is quickly captured and degraded by the human immune system via the reticuloendothelial system, driven by red pulp macrophages in the spleen and Kupffer cells in the liver [146]. Complement, antibodies, and other substances opsonize viral particles, enhancing endothelial cell and macrophage engagement and phagocytosis. There have been no instances of inactivated particles reverting virulence or causing poor dose tolerance to oncoviral treatment. In oncoviral therapy, managing the level of local immunosuppression is a difficult task.

On the one hand, immunosuppression can improve the therapy's intratumoral distribution. In contrast, enhancing the host immune system will improve transfected tumor cell targeting while reducing intratumoral viral dissemination [147]. As a result, and to date, the only way to deliver oncoviral therapy in sufficient quantities to be clinically effective is through locoregional or direct inoculation. Furthermore, image-guided delivery is inextricably linked to oncoviral therapy's potential effectiveness and broad use. The idea of image guiding is broad, encompassing planning, aiming, managing, evaluating, and analyzing treatment reaction for lesions, all of which are critical to the therapy's success. Image evaluation for planning is vital not only for detecting neoplastic tumors, but also for identifying and selecting therapeutic delivery targets. Detecting a large but necrotic lesion, for example, may be preferable to identifying a smaller but active metabolism/proliferation lesion. Functional cells exhibit central roles in viral transfection and immune cell activation, and these tissues could also be collected for tumor reaction monitoring. Moreover, the needle path anticipated can be examined to ensure that it does not cross any inappropriate or high-risk anatomical features. Also, image guidance offers direct access to certain body parts which would otherwise be inaccessible to effective hematogenous distribution of systemic therapy, including tumors with low mitotic indices or malignancies with poor vascularization. According to clinical research, oncolytic viruses as a monotherapy are less likely to obtain optimal therapeutic results. On the other hand, oncolytic viruses appear to be strong candidates for combining with other therapies, particularly immunotherapy. Furthermore, to verify the biosafety of oncolytic virotherapy, additional clinical trials are required, and further OVs should be developed as quickly as feasible to be used in clinical treatment. Researchers will perform novel combination therapies with other drugs in the future, as well as new genetically altered OVs and delivery systems. In cancer treatment, OVs will be the most effective therapeutic option once physical barriers, immunosuppressive milieu, and host removal of OVs are eliminated.

## Figures and Tables

**Table 1:** Clinical trials of OV therapy in various cancers.

Oncolytic virus	Combination	Phase	Status	Trial no.	Type of cancer	Ref.
Vaccinia virus	Flucytosine	2	Recruiting	NCT04226066	Solid tumors	[135]
Vaccinia virus	Durvalumab Tremelimumab	2	Active	NCT03206073	Colorectal cancer	[136]
Adenovirus	Gemcitabine Abraxane®	1	Completed	NCT02045589	Pancreatic cancer	[137]
Adenovirus	HER2-specific CAR T cells	1	Recruiting	NCT03740256	Solid tumors	[138]
Adenovirus	Pembrolizumab	2	Completed	NCT02879760	NSCLC	[139]
Adenovirus	Temozolomide	1	Completed	NCT01956734	Glioblastoma	[140]
Herpes virus	Ipilimumab	2	Completed	NCT01740297	Melanoma	[141]
Herpes virus	Pembrolizumab	3	Terminated	NCT02263508	Melanoma	[142]
Reovirus	Paclitaxel carboplatin	2	Completed	NCT00984464	Melanoma	[143]
Reovirus	Gemcitabine	2	Completed	NCT00998322	Pancreatic cancer	[144]
Reovirus	Paclitaxel	2	Completed	NCT01656538	Breast cancer	[145]

## Data Availability

All data generated or analyzed during this study are included in this published article.

## Abbreviations

OV: Oncolytic viruses
HSV-1: Human herpes simplex virus I
APC: Antigen-presenting cell
DC: Dendritic cell
NDV: Newcastle disease virus
NK: Natural killer
TAM: Tumor-associated macrophage
MDSC: Myeloid-derived suppressor cell
VEGF: Vascular endothelial growth factor
ICI: Immune checkpoint inhibitor
CTLA-4: Cytotoxic T-lymphocyte-associated protein 4
PD-1: Programmed death 1
PD-L1: Programmed cell death ligand 1
TIGIT: T cell immunoreceptor with Ig and ITIM domains
MV: Measles virus
VSV: Vesicular stomatitis virus
MMP3: Matrix metalloproteinase-3
CAR-T: Chimeric antigen receptor T
MHC: Major histocompatibility complex
ICD: Immunogenic cell death
TIL: Tumor-infiltrating leukocyte.
